# Editorial: 3D Cell Culture Systems for Cardiovascular Tissue Engineering: *In vitro* Modelling and *in vivo* Regenerative Therapies

**DOI:** 10.3389/fcvm.2021.675676

**Published:** 2021-06-17

**Authors:** Sanika Joshi, Won Hee Lee, Pu Chen, Vahid Serpooshan, Huaxiao Yang

**Affiliations:** ^1^Biomedical Engineering, University of North Texas, Denton, TX, United States; ^2^Texas Academy of Mathematics and Science, University of North Texas, Denton, TX, United States; ^3^Department of Basic Medical Sciences, University of Arizona College of Medicine, Phoenix, AZ, United States; ^4^Department of Biomedical Engineering, Wuhan University School of Basic Medical Sciences, Wuhan, China; ^5^Department of Biomedical Engineering, Emory University School of Medicine and Georgia Institute of Technology, Atlanta, GA, United States

**Keywords:** 3D culture, cardiac patches, human cardiac muscle patches, induced pluripotent stem cell-derived endothelial cells, disease modeling, organoids

Although 3D cell culture systems have become an emerging need in the field of cardiovascular regenerative medicine, they are not yet as widespread as conventional 2D cell culture systems, which use monolayers of cells in Petri dishes. The conventional 2D cell culture is not effective or accurate in its response to *in vivo* drugs, toxins, or signaling modifiers (1). This is because the cellular microenvironment in a 2D culture is significantly different from that in native heart tissue. Cardiac extracellular matrix (ECM) is composed of a variety of proteins and biomechanical cues, influencing cardiovascular diseases (CVDs) and regeneration. Compared with a 2D culture, 3D cell culture systems more closely replicate these *in vivo* situations and benefit from the advent of various new bioengineering technologies (2).

In view of the heart's limited ability to renew itself, various strategies of myocardial tissue engineering and repair have attracted growing attention. As reviewed by Jarrell et al., there are three challenges to achieving *in situ* tissue engineering of cardiovascular systems: (1) recapitulation of the myocardial tissue architecture; a primary consideration for engineering is to make clinically useful tissues such as the architecture of scaffolds, which entails the arrangement of the ECM; (3) (2) tissue vascularization; since the myocardium is precisely organized, enervated, and populated with cardiomyocytes (CMs), endothelial cells (ECs), cardiac fibroblasts (CFs), and resident macrophages to allow high metabolic activity and hypertrophy, tissue vascularization is another crucial consideration; (4, 5) (3) immune system modulation; host immune response must also be reviewed to avoid immune rejection during the integration of engineered cardiac tissues. Moreover, Jarrell et al. additionally provide specific strategies for the generation of tissue-engineered myocardial patches.

Cardiac patches are functioning pieces of heart tissue grown in the laboratory and used to replace damaged CMs before the whole heart is irreversibly remodeled by the resulting fibrotic scar tissue (6). Mei and Cheng provide a review of the therapeutic ingredients and scaffold biomaterials, the two components of cardiac patches, along with a discussion of clinical applications. For a variety of cell sources—e.g., cardiac stem/stromal cells, mesenchymal stem cells (MSCs), and induced pluripotent stem cells (iPSCs)—cardiac patches enhance the cellular retention and survival ratio. Acellular patch devices demonstrate a more direct therapeutic role in heart repair with the release of growth factors, extracellular vesicles, and miRNAs. Patches without biomolecules present a passive restraining function to protect the injured myocardium (Mei and Cheng). Materials for patch fabrication, from natural to synthetic polymers, are chosen in consideration of biocompatibility, biodegradability, and mechanical strength. Challenges and future directions for clinical applicability include (i) minimally invasive delivery of cardiac patches, (ii) an improvement in biocompatibility, and (iii) long-term cell storage (Mei and Cheng).

Wang et al. contribute to this Research Topic by elaborating an application of these cardiac patch devices for the prevention of post-infarction left ventricular (LV) remodeling. Human cardiac muscle patches (hCMPs) are an effective alternative to transplanted cells in supporting the injured myocardium. However, they lack enough vascularization to support the heart's metabolic activity and thus are limited in thickness (7). Since hCMPs are meant to replicate the native, multicellular structure of heart tissue, they are often biofabricated using CMs, ECs, smooth muscle cells (SMCs), CFs, iPSC derivatives, progenitor cells, and spheroids (Wang et al.). Cell sheets, decellularized ECM, 3D printing technologies, the cells containing “bioinks,” and computer-aided design mark some of the advancements from the traditional cardiac tissue manufacturing (Wang et al.). Wang et al. add a comparison of invasive vs. non-invasive hCMP delivery methods and large animal models vs. small animal models for hCMP testing. For future clinical applications of hCMPs larger, thicker, and more vascularized constructs are essential through the advanced tissue engineering approaches of layer-by-layer assembly, *in vitro* perfusion, and engineered vascular networks.

Aside from the cardiac patch, the applications of iPSC-derived ECs also show great promise for the development of *in vitro* cardiovascular disease models and regenerative therapies (Kennedy et al.). To test endothelial dysfunction, which can cause CVDs such as atherosclerosis and hypertension, it is difficult to source primary ECs directly from the patients. On the other hand, iPSCs, which can be renewed almost indefinitely, can be used to differentiate into ECs through the development from mesoderm to vascular endothelium and arterial/venous specification. Kennedy et al. detail the methods of vascular iPSC-EC differentiation and characterization using an embryoid body, the addition of small molecules, and selective transcription factor expression. The authors also review the applications of 3D iPSC-derived endothelium in disease modeling, drug screening, and therapeutic applications in the forms of microphysiological systems, organoids, and cell therapies. Challenges to these applications include the need to improve organoid vascularization, maturations, and tissue specificity (8).

The use of iPSCs for disease modeling also extends to congenital heart disease (CHD). CHDs are structural abnormalities that present at birth, which disproportionately affect minorities as a result of differences in genetics and environment. iPSCs, omics, and machine learning technologies are used to investigate such race-specific genetic variants and the cause of CHD's higher incidence in minorities (Mullen et al.). Mullen et al. review the prevalence, modifiable and non-modifiable risk factors, and genetics of CHDs. The advances of genetic and iPSC technologies provide a unique opportunity to both identify and address the causes of racial disparities in CHD. With the use of identified genetic variants, CHD modeling systems, and genome editing technology, more effective treatments within a clinical setting are within reach (Mullen et al.).

The advancement of 3D histotypic and organotypic cultures has no doubt spurred cardiovascular studies. Engineering heart tissue and muscle with new technologies—such as nano-biomaterials, microfluidics, 3D (bio)printing, bioassembly, stem cells, genome editing, high-content screening, bioimaging, and next-generation omics—is a great leap forward from the more traditional use of 2D cell culture. Although there are still challenges for the clinical translation of such technologies, the potential benefits of the convergence between 3D cell culture and bioengineering technologies paves the way for a better understanding of the cardiovascular disease, development, and potential therapies.

## Author Contributions

All authors listed have made a substantial, direct and intellectual contribution to the work, and approved it for publication.

## Conflict of Interest

The authors declare that the research was conducted in the absence of any commercial or financial relationships that could be construed as a potential conflict of interest.
